# Aluminium as a Base for Artificial Teeth

**Published:** 1870-08

**Authors:** J. A. Hodgen

**Affiliations:** Lexington, Ky.


### ARTICLE V.

### *Aluminium as a Base for Artificial Teeth.*

By J. A. Hodgen, Lexington, Ky.

Several Dentists wishing to know my experience in the use of aluminum as a base for artificial teeth, I give it with pleasure, though it is not so extensive as I could wish.

I have been using it for more than a year, and have many patients wearing it who are well pleased with it. I wore it myself in an upper plate for a few weeks. I could feel or taste no difference between it and gold.

Its qualities are such that the secretions of the mouth have no effect on it, so far as my observation extends.

The acid contained in the plaster, in vulcanizing, has less effect on it than on twenty-carat gold. It is tasteless, and a good conductor. It is very easily manipulated with plates, being softer than gold or silver, it strikes up well. I have some objections to it, however, the chief of which are: first, it is hard to polish; second, difficult to solder; third, not quite so hard as we could wish.

The first objection can be overcome by a little more industry and labor than for gold and rubber plates. The second can be remedied by using the rubber attachment or the star solder, or casting on the teeth with adamantine. The latter plan I prefer for lower sets, as we want them heavier than rubber plates are generally made. For partial sets, I use the star solder or my own, either answers a good purpose. The cast attachment will make some upper sets too heavy in cases that have lost much of the alveolus, and when we wish to restore its shape. The third objection I hope will be alleviated by some addition to the aluminum to harden it. Silver will do it, but it is not pure enough and it makes it more brittle; yet I think it will do much good in a small proportion

This, however, is not much objection to it. When it is struck up and left unannealed, it is much harder and stiffer. When we want clasps in partial sets, I have the plate thick, to give strength to them—18 or 20, of our common gauge. It swedges up much easier than gold or silver, and annealing makes it much softer.

As to a comparison between it and rubber, I regard the former as being far superior; for, whereas the rubber is brittle and easily broken, and filthy by absorption and slow decay, the aluminum is strong, pure, tasteless and a good conductor. We know that rubber is a poor conductor. This must have the effect, in some cases, by the retention of a superabundance of heat, to produce inflammation of the mucus membrane underneath the plate.

I have examined a great number of cases within the past several years, and my observation is that we can see some traces of inflammation, in about nine cases out of ten, in the membrane, under the plate. I have had several cases where the inflammation was so great as to produce intense redness, thickening of the membrane, and smarting and burning under the plate; also a few cases with all these symptoms attending the use of lower plates. I feel satisfied, partly from this last circumstance, that the nonconducting properties of the rubber are not the only cause of this inflammation, as the space covered by the rubber on the lower jaw is so narrow that the heat can readily escape. We must, therefore, look for the cause elsewhere.

Now, when we consider that the rubber is so largely composed of sulphuret of mercury—one third, I believe—and worn constantly in contact with so delicate aud sensitive a membrane, it is marvelous that there is not more mischief done.

I find again, in some cases, a great paleness—as if scalded—of the membrane, directly under the plate, showing loss of vital action.

I am glad to see that some Dentists are occasionally holding up these effects of the rubber to the public, that they may take warning not to use it in plates for artificial teeth.

If it be true that the rubber has such deleterious effects on our patients, is it not morally our duty to desist from making it and substitute something better? But they are so easily made, and the material so cheap, it will be a difficult thing to get all the Dentists to quit making it. Many there are who only know how to make these, and to think of changing to metal plates that require so much more labor, cost and skill, they will hardly be persuaded, against their wills. But, to aid as much as I can in a reform move, I will give, in my next, with more minuteness, my plan of manipulating the aluminum into plates, with my improved die metal, &c. with this metal it is easy to get a plate ready to try on in forty or fifty minutes after the impression is taken.—*Dental Register*.

